# Treatment of Eczema

**Published:** 1870-03

**Authors:** 


### Treatment of Eczema.

Dr. Erasmus Wilson, F. R. S., (*Journal of Cutaneous Medicine*), after endeavoring to show in the treatment of eczema, that the patient needs repose, quiet, rest, and proper and nutritious food without stint or limit, asks: "What else is to be done? Shall we purge or physic the patient? God forbid. If the bowels are confined, help them with a little magnesia and rhubarb, or manna, or castor-oil; if the patient is emaciated, and exhibits a tendency to waste, the prostration may be checked by cod-liver nerve-restorer, arsenic. Try two minims of the liquor arsenicalis three times a day in the following combination:—℞ Vini ferri, ℥ iss.; syrupi simplicis, ℥ iij.; liquoris arsenicalis, ʒ i.; aquæ anethi, ℥ij. Misce. One drachm will give two minims of liquor arsenicalis. It may be administered pure, and just at the end of the meal, so as to be mixed with the meal in the stomach. The physician should watch narrowly the arsenical solution to see that it occasions no nausea, no gripes, and no prostration of power. These are its primary effects when it disagrees with the system, and there are sundry subsequent effects, such as a puffy swelling of the eyelids, the cheeks, or limbs; and later still, congestion of the vessels of the conjunctiva.—*Medical Record.*

